# Structural Basis of Parasitic HSP90 ATPase Inhibition by Small Molecules

**DOI:** 10.3390/ph15111341

**Published:** 2022-10-29

**Authors:** Giusy Tassone, Marco Mazzorana, Cecilia Pozzi

**Affiliations:** 1 Department of Biotechnology, Chemistry and Pharmacy, Department of Excellence 2018–2022, University of Siena, Via Aldo Moro 2, 53100 Siena, Italy; 2 Diamond Light Source Ltd., Diamond House, Harwell Science & Innovation Campus, Didcot OX11 0DE, UK

**Keywords:** heat shock proteins, HSP90, protozoan parasites, neglected tropical diseases, inhibitors, selectivity, chaperone function

## Abstract

Protozoan parasites are responsible for several harmful and widespread human diseases that cause high morbidity and mortality. Currently available treatments have serious limitations due to poor efficiency, strong adverse effects, and high cost. Hence, the identification of new targets and the development of specific drug therapies against parasitic diseases are urgent needs. Heat shock protein 90 (HSP90) is an ATP-dependent molecular chaperone that plays a key role in parasite survival during the various differentiation stages, spread over the vector insect and the human host, which they undergo during their life cycle. The N-terminal domain (NTD) of HSP90, containing the main determinants for ATPase activity, represents the most druggable domain for inhibitor targeting. The molecules investigated on parasite HSP90 are mainly developed from known inhibitors of the human counterpart, and they have strong limitations due to selectivity issues, accounting for the high conservation of the ATP-binding site between the parasite and human proteins. The current review highlights the recent structural progress made to support the rational design of new molecules able to effectively block the chaperone activity of parasite HSP90.

## 1. Introduction

Obligate protozoan parasites belonging to the kinetoplastid and apicomplexan kingdoms are the etiological agents of various threatening neglected tropical diseases (NTDs), including human African trypanosomiasis (HAT, also known as sleeping sickness), Chagas disease, different forms of leishmaniasis, toxoplasmosis, and malaria [1,2]. The kinetoplastid *Trypanosoma brucei* (*T. brucei* or *Tb*) is responsible for HAT, *Trypanosoma cruzi* (*T. cruzi* or *Tc*) for Chagas disease, and *Leishmania* spp. for various forms of leishmaniasis, spanning from cutaneous to fatal visceral infections [3]. On the other hand, toxoplasmosis and malaria are due to the apicomplexan *Plasmodium falciparum* (*Pf*) and *Toxoplasma gondii* (*Tg*), respectively [4,5]. NTDs are endemic in several countries, especially in tropical and subtropical areas. Recently the WHO estimated billions of people worldwide to be at risk of infections from these parasites, as millions of NTDs cases are counted every year [1,6]. In the future, the incidence of these diseases is expected to increase due to various factors, including climate change and widespread human migration. This will exacerbate public health issues and related economic burdens [7,8]. Current therapeutic regimens for NTD treatment are limited and characterized by poor efficacy, high toxicity, and rapid insurgence of resistance [9,10,11]. Furthermore, the low investments in research and drug development towards NTDs make this scenario even more worrying [6]. Despite the important steps taken in the treatment of parasite diseases achieved in the recent years, problems such as difficulties in administration and occurrence of adverse side effects are still unresolved [12,13,14]. Hence, the development of novel effective drugs against protozoan parasites is an urgent need.

The life cycle of protozoan parasites involves various stages of differentiation occurring within two systems, namely, the insect vector and the mammalian host (comprehensive parasite life cycles are available at https://www.cdc.gov/parasites/index.html; access date: 1 September 2022) [15]. Parasites encounter extremely different physiological conditions in insect and human hosts, such as pH, temperature, and nutrient supply, affecting the structural and functional integrity of their cellular components [16,17,18,19]. Among these environmental factors, temperature change is a major cellular stress that parasites face in adapting to mammalian conditions, which are overcome through the synthesis of heat shock proteins (HSPs) [20,21,22]. HSPs are a family of chaperones that contribute to the survival of parasites under stress conditions by promoting protein integrity and cell homeostasis [20,21,22]. Within this protein family, HSP90 is known to play a pivotal role in the survival and proliferation of protozoan parasites, being involved in different stages of their life cycle [20,21,23,24,25,26]. Over the last decades, HSP90 has been widely investigated as a target for anti-parasitic drugs [23,27,28,29,30,31,32]. This review aims to summarize the current structural and functional understandings of protozoan parasite HSPs, with a particular focus on HSP90 and the recent progress in the development of inhibitors targeting this protein.

## 2. Protozoan Parasite HSPs and HSP90

HSPs are ubiquitous molecular chaperones whose main role is to stabilize nascent proteins in a folded and active state. They are also involved in key cellular functions, including regulation of gene expression, signaling cascades, protein degradation, and stress resistance [33,34]. HSPs are classified according to their sequence homology and molecular weight into six families: small-molecular weight HSPs (sHPSs), HSP40, HSP60, HSP70, HSP90, and HSP100 [33,34,35]. sHSPs are characterized by molecular weights ranging from 12 kDa to 43 kDa and by a peculiar tendency to form large globular oligomers with sizes exceeding 800 kDa [35,36,37,38]. These proteins share a conserved α-crystallin domain consisting of ~90 residues. Although their cellular functions are not yet fully understood, sHSPs have been proposed to play a role in stress adaptation and parasite development in the mammalian host [35,36,37,38]. The HSP60 family consists of two groups of proteins, known as chaperonin 10 and chaperonin 60, which share similar architectures despite being unrelated in their primary structures [39]. These proteins are over-expressed under stress conditions and contribute to the refolding of damaged or misfolded proteins [40,41]. The HSP70 family members play a pivotal role in various fundamental cellular processes, including folding and assembly of newly synthesized proteins, signal transduction, cycle regulation, differentiation, and death [32]. HSP70s cooperate with their co-chaperone HSP40, also known as J protein, for the presence of a 70-amino acid J domain [42]. This accessory protein is an obligate partner of HSP70, enabling substrate recruitment and stimulating the ATPase activity of HSP70 [43,44,45,46,47]. The AAA+ chaperones of the HSP100 family mainly contribute to unfolding and disassembly of protein aggregates and polypeptide complexes. They are arranged in hexameric barrels, which recruit their substrates through the HSP70–HSP40 complexes [48,49,50].

The last family is HSP90, a chaperone known to mediate folding, stabilization, and assembly of nascent protein chains [20,21,22]. Five different isoforms of HSP90 have been identified in eukaryotes: constitutive and inducible cytosolic HSP90s, an endoplasmic reticulum glucose-regulated protein 94 (Grp94), mitochondrial tumor necrosis factor receptor-associated protein 1 (TRAP1), and membrane-associated HSP90N [51,52]. Among the different organisms, orthologues of the cytosolic HSP90 have been identified and classified according to their relative molecular weight (HSP82, HSP83, HSP84, HSP86, and HSP89). In parasites, cytosolic HSP90 (alternatively referred to as HSP83) plays a pivotal role in various phases of the life cycle and in pathogenicity. The parasites *L. donovani, L. mexicana, L. amazonensis, T. gondii*, *T. brucei*, *T. cruzi*, and *P. falciparum* require HSP83 to withstand environmental stresses such as pH and temperature fluctuations in the human host [20,21,24,25,26,53,54,55]. HSP90 acts on native-like proteins or client proteins with the assistance of a large variety of co-chaperones (vide infra). The function of HSP90 is carried out using ATP hydrolysis as a source of energy for the folding of client proteins and for preventing their aggregation [56]. The ATPase function of HSP90 is crucial, and its blocking leads to client protein degradation and cell death [57].

## 3. HSP90 Structure

**Figure 1:**
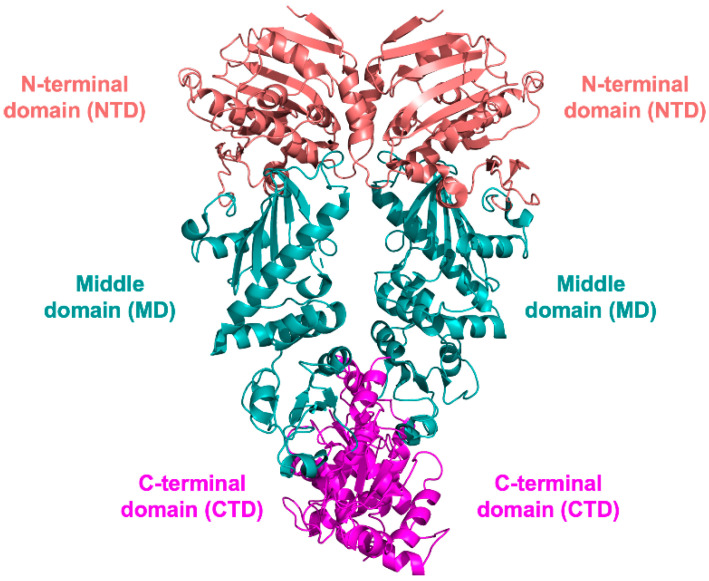
Cartoon representation of the dimeric full-length *Saccharomyces cerevisiae* HSP90 in complex with the cochaperone p23 and the ATP analogue adenosine-5-(β,γ-imido)triphosphate (AMP-PNP; PDB id 2CG9 [58]). The NTD is colored coral, the MD cyan, and the CTD magenta.

In its active form, HSP90 is a homodimer in which the two monomers are arranged in parallel and linearly to each other in the shape of a left-handed helical twist around its long axis [34,58]. Each monomer consists of three conserved domains, namely, the N-terminal (NTD, ~25 kDa), the middle (MD, ~35 kDa), and the C-terminal (CTD, ~20 kDa) domains (Figure 1), each performing specific functions.

The NTD contains the main structural elements required for ATP binding, but it is unable to perform ATP hydrolysis as an isolated domain [59,60]. Indeed, NTD dimerization and the contribution of structural elements belonging to the MD are required for ATPase activity [59,60]. The MD contributes to modulate the ATPase activity by binding the substrate γ-phosphate [61]. The NTD and MD are connected by a disordered region mainly consisting of charged amino acids and thus are referred to as the charged linker [62]. Previous studies have proven the contribution of this linker to protein dimerization and regulation of ATPase activity [63] (*vide infra* Section 4.3). The CTD is responsible for the inherent dimerization of the protein [64] and allosteric regulation. All three domains are involved in the recognition and binding of co-chaperones and client proteins. A fundamental requisite for the chaperone function of HSP90 is its high flexibility (*vide infra* Section 4.3), a peculiarity that has hampered the structural characterization of the whole protein. Indeed, only a few structures of full-length eukaryotic proteins [58,65] have been reported so far, and none of them is of the protozoan parasite HSP90s, currently studied only as isolated domains.

### 3.1. NTD Structure

**Figure 2:**
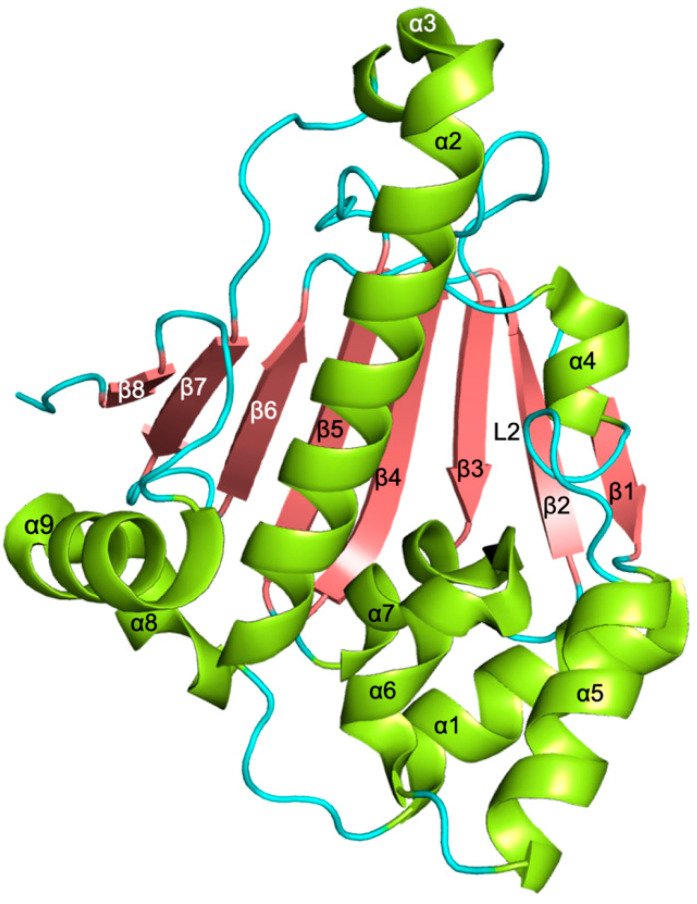
Cartoon representation of the tertiary structure of *P. falciparum* HSP90-NTD (ligand-free state; PDB code 3K60 [66]). The α-helices are colored light green, β sheets coral, and loops cyan.

The NTD is structured as a two-layer sandwich, formed by a β-sheet (stands β1–β8) on one side and a cluster of nine α-helices on the other, delimiting the ATP binding pocket, a flat-bottomed cone ~15 Å in depth (Figure 2).

**Figure 3:**
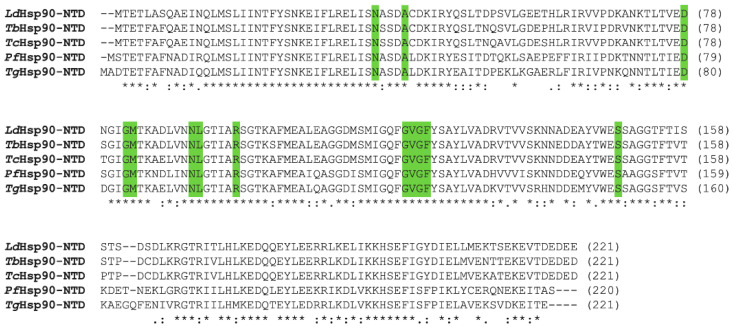
Sequence alignment among the HSP90-NTDs of various parasites (UniProtKB codes *Ld*HSP90: F1CCS2; *Tb*HSP90: Q389P1; *Tc*HSP90: P06660; *Pf*HSP90: Q8IC05; *Tg*HSP90: Q7Z1F7) performed with Clustal Omega. Amino acids involved in ATP binding are highlighted in green. Conserved residues are indicated by asterisks, whereas residues with similar and weakly similar properties are indicated by colons and dots, respectively (below the residues).

One side of the β-sheet is mostly hydrophobic and is packed against a wall of five α-helices. Four of these helices (α1, α2, α4, and α9) are parallel to the β-sheet, while the fifth (α7) is placed at its periphery, being almost perpendicular to it. A second set of two helices (α5 and α6) is located between the former layer and the two shorter helices (α3 and α8). The ATP-binding site is defined by the central part of the β-sheet, the α4–α5 loop (loop L2), and helices α2, α4, and α7. The region included between helices α4 and α6 is highly flexible, and specifically loop L2 can assume two main conformations, named “open” and “closed”, related to the capacity of accommodating the substrate in the ATP-binding site. In fact, when loop L2, usually far from the active site, approaches the catalytic pocket to give the “closed” state, it buries the ATP-binding pocket, making it inaccessible to the nucleotide. Thus, the transition from the “closed” to the “open” state is required for the formation of the complex between HSP90 and ATP. Intermediate conformations have been observed in the structures of parasite HSP90-NTDs, supporting the functional role of the loop L2 as an active site lid during substrate binding [66,67]. The amino acid composition of the ATP binding pocket is highly conserved among protozoan parasite NTDs, as highlighted by the sequence alignment shown in Figure 3.

**Figure 4:**
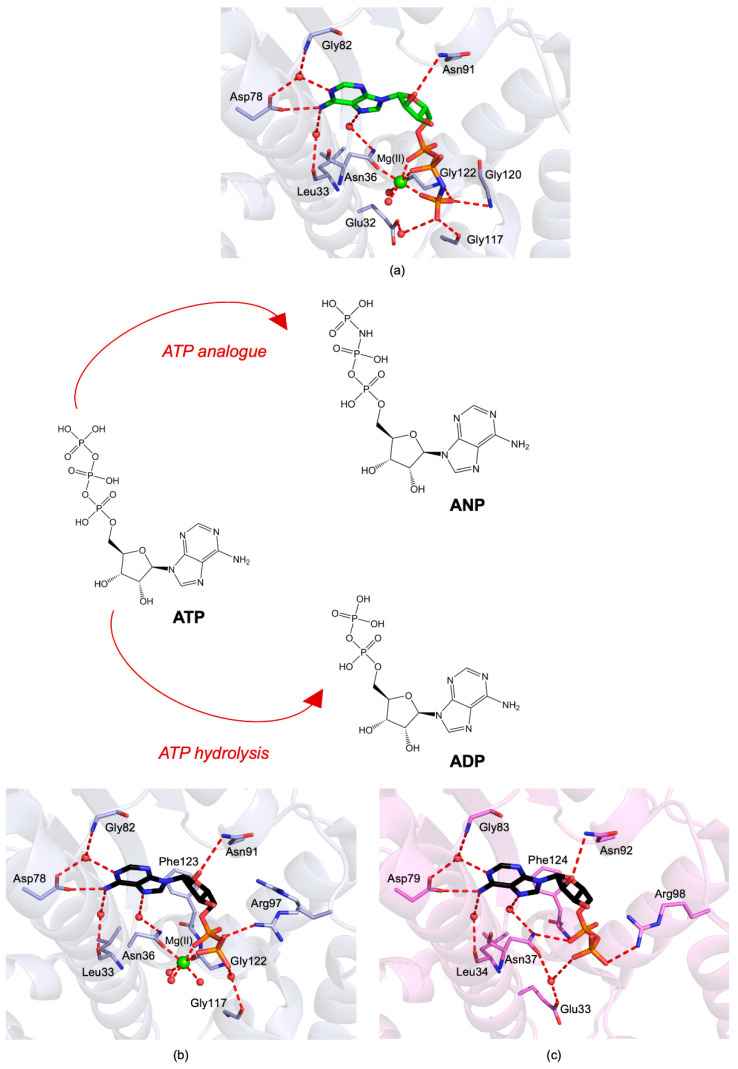
Active-site view of (**a**) *Lm*-NTD (light blue cartoon and carbons) in complex with phosphoaminophosphonic acid–adenylate ester (ANP, in sticks, green carbons; PDB id 3H80, unpublished results); (**b**) *Lm*-NTD (light blue cartoon and carbons) in complex with ADP (in sticks, black carbons; PDB id 3U67, unpublished results); (**c**) *Pf*-NTD (violet cartoon and carbons) in complex with ADP (in sticks, black carbons; PDB id 3K60 [66]). In all figures, hydrogen bonds are displayed as red dashed lines; the Mg(II) ion and water molecules are displayed as green and red spheres, respectively; oxygen atoms are colored red, nitrogen blue, and phosphorus orange.

To date, no crystal structures have described the binding of the physiological substrate ATP to the NTD protozoan parasite HSP90s. Nonetheless, structural information for *Lm*-NTD both in its pre-catalytic state (with the non-hydrolysable ATP analogue ANP, PDB id 3H80, Figure 4a) and bound to ADP (PDB id 3U67 [67], Figure 4b) and *Pf*-NTD in complex with ADP (PDB id 3K60 [66], Figure 4c) is available. In all of them, the adenosine-binding pocket shows several interactions with the ligand. In particular, Asp78 forms contacts both directly and through a network of water molecules [68]. Furthermore, water-mediated interactions occur between the ligand and the residues Asn91, Gly122, Phe123, and Asn36. These perfectly match the interactions observed with in the ADP-bound structure of *Pf*-NTD (Asp79, Asn92, Gly121, Phe124, and Asn37).

The catalytic Mg(II) ion coordinates the α and β phosphate groups of ADP in the structure of *Lm*-NTD (Figure 4b). The octahedral coordination of the Mg(II) ion is completed by the Asn51 amide oxygen and by three water molecules. This ion is absent in the structure of *Pf*-NTD (Figure 4c), but a reprocessing of data through PDB-redo [69] correctly displays the unaccounted electron density bridging the β phosphate with the side chain of Asn37 (Figure S1).

Although no crystal structure of parasite HSP90-NTD bound to the natural substrate (ATP) is available, the interactions between *Lm*-NTD and ANP resemble those in the ATP-bound structure of human HSP90-NTD (PDB id 3T0Z [70]). Here, the γ-phosphate of ANP in complex with *Lm*-NTD (Figure 4a) forms a water-mediated interaction with Glu32, and it is H-bonded with the Gly117 backbone carbonyl and the Gly120 and Gly122 backbone nitrogens. The most striking change is the repositioning of Arg97, which, during hydrolysis, moves from a distal position to the proximity of the β phosphate moiety.

### 3.2. MD and CTD Structure

**Figure 5:**
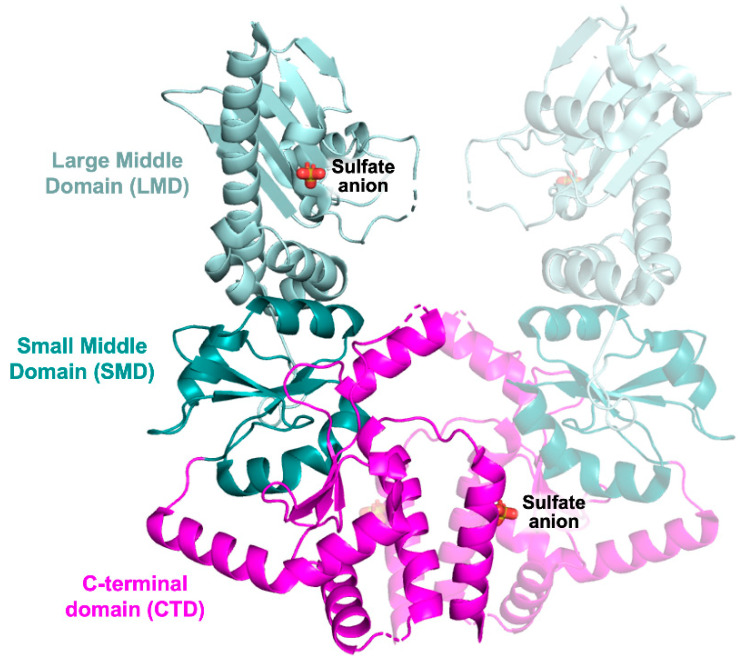
Crystal structure of the *Lm*-MD–CTD dimer (PDB id 3HJC, unpublished results). The partner subunit, generating the protein dimer, is represented in transparency. The LMD is colored light cyan, the SMD cyan, and the CTD magenta. Sulfate anions are displayed in sticks.

The structure of the MD includes the large middle domain (LMD) linked through three short helices to the small middle domain (SMD) (Figure 5). The LMD has a three-layer α–β–α structure, whereas the SMD has an α–β–α sandwich fold.

The CTD is folded as a three stranded β-sheet sandwiched between two sets of α-helices (Figure 5). The CTD is responsible for constitutive HSP90 dimerization mediated by the two carboxyl-terminal helices of the facing protomers forming a four-helix bundle at the dimer interface. To date, only one structure of protozoan parasite HSP90-MD and CTD has been reported in the PDB, namely, the *Lm*-MD–CTD structure (PDB id 3HJC, unpublished results). The CTD is connected to the SMD through an extended amphipathic loop. Two sulfate anions are present in the structure, one placed at the CTD dimer interface and the second in the LMD, bound to two arginine residues exposed on the protein surface (Figure 5).

## 4. The HSP90 Machinery: Co-Chaperones, Clients, and Mechanism of Action

### 4.1. Co-Chaperones

Co-chaperones are generally defined as proteins interacting with HSP90 and assisting its function without relying on it for their own folding and stability [71]. A plethora of co-chaperones has been identified, representing the most important layer of regulation to HSP90 chaperone function. All three domains harbor sites for the interaction with co-chaperones that may bind either in combination or in antagonism with each other [72]. Through this complex network of interactions, co-chaperones modulate HSP90 activity, playing roles at different stages of the ATPase cycle and tuning both catalytic activity and client specificity. Notwithstanding their structural heterogeneity, some common motifs in HSP90 co-chaperones allow a categorization in two major classes: the tetraicopeptide repeat (TPR) domain-containing and the non-TPR-containing co-chaperones [56,71]. This grouping relies on the presence or absence of an α-helical TPR domain capable of interactions with the conserved MEEVD carboxy-terminal sequence of cytosolic HSP90s. A complete up-to-date list of HSP90 co-chaperones is available at http://www.picard.ch/downloads/HSP90interactors.pdf (Access date: 1 September 2022) [71,73]. At variance with cytosolic HSP90, whose co-chaperones have been better characterized, the compartmentalization of other isoforms, such as the ER Grp94 and the mitochondrial TRAP1, has strongly limited the identification of their co-chaperones (data still lacking for parasite proteins). The co-chaperone repertoire of HSP90 is often incomplete in parasites with respect to the human host, with some homologs of human co-chaperones (e.g., CDC37) missing (or still not identified) in many protozoa [51,54,71,73]. More efforts are needed to deeply investigate parasite co-chaperones and their role on HSP90 activity. A further interesting point is to investigate how the omission of these co-chaperones in these parasites impacts on the HSP90 cycle. This information will provide key insight to unveil the mechanism of action of HSP90 in protozoa parasites.

### 4.2. Client Proteins

The partners interacting with HSP90 and needing its chaperon activity to acquire and keep their active state are referred to as client proteins [51,74,75,76]. Several HSP client proteins are central hubs in complex biological pathways and cascades, making the HSP90 activity a pivotal factor in cell regulation. To date, more than 400 client proteins, implicated in protein folding and degradation, signaling pathways, cellular trafficking, cell cycle regulation, differentiation, and other functions, have been identified. A comprehensive, revised list of HSP90 clients can be found at http://www.picard.ch/downloads/HSP90interactors.pdf (Access date: 5 September 2022) [71,73]. Historically, HSP90 clients included only proteins constitutively requiring HSP90 to maintain their functional state. Nonetheless, a recent study, relying on multiplexed proteome dynamics profiling, drastically expanded the set of clients, identifying a remarkably high number of proteins transiently exploiting HSP90 during their de novo synthesis [74]. The transient nature of their interactions with HSP90 hinders their recognition as client proteins; nonetheless, the knowledge of HSP90 interactors (both clients and co-chaperones) is of crucial importance to estimate the potential cellular effects of treatments inhibiting HSP90 activity.

### 4.3. Mechanistic Insight on Parasite HSP90

**Figure 6:**
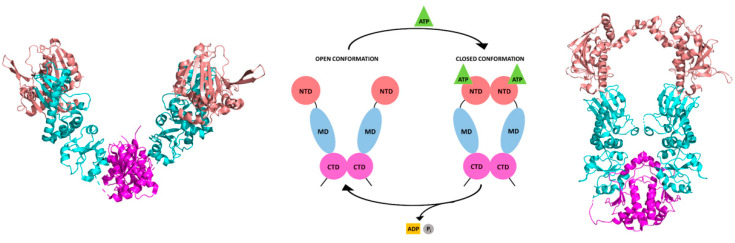
Schematic representation of the ATPase cycle of HSP90. The open and closed conformations are depicted. Since no structural information is available on full-length parasite HSP90, the rendering of the structures of the open and closed states of HSP90 has been done on a representative protein, *Escherichia coli* HSP90. To the left, the cartoon representations of the dimeric full-length *Escherichia coli* HSP90 in the open state (PDB id 2IOQ [80]) and in the closed state in complex with ADP (PDB id 2IOP [80]) to the right are shown. The NTD is colored coral, the MD cyan, and the CTD magenta.

HSP90 is a molecular machine that interacts with client proteins and promotes their folding through conformational changes regulated by its ATPase activity and by highly specific protein–protein interactions [51]. Indeed, HSP90 activity and conformational dynamics are largely modulated by ATP binding, mainly mediated by the NTD of the protein [51,77]. To perform ATPase activity, the protein undergoes significant conformational changes accounting for the rearrangement of both NTD and MD (Figure 6). The rearrangement of these domains is essential to weaken the bond between the β- and γ-phosphates of ATP, orienting the latter proximal to a highly conserved arginine residue of the MD and triggering its hydrolysis [51,61]. The charged flexible loop linking the NTD to the MD plays a fundamental role in modulating interdomain communication to perform the chaperone function [78].

The HSP90 cycle start by ATP binding to the NTD, inducing the ATP lid to close over the nucleotide, followed by a sequence of structural rearrangements that lead to the dimerization and swapping of the NTD (closed state, Figure 6) [51,61,79]. In general, all HSP90s bound to ATP can associate with unfolded/partially folded client proteins to perform their chaperone activity. Then the MD approaches the NTD, altering the position of the MD catalytic loop and triggering ATP hydrolysis. After ATP hydrolysis, the cycle is completed by ADP and phosphate release and client protein dissociation, allowing HSP90 to return to the open conformation (Figure 6), characterized by NTD dissociation. This general model for the ATPase cycle is applied to all HSP90 paralogs and isoforms. Although isoform-specific differences exist, the lack of structural information on parasite HSP90s prevents their peculiar definition in these organisms.

## 5. HSP90 of Protozoan Parasites as Drug Target

HSP90 performs its chaperone function on client proteins using the energy generated from ATP hydrolysis. Since the ATPase activity occurs at the NTD level, molecules able to block its active site by competing with ATP act as HSP90 inhibitors. Client proteins that fail to correctly fold end up in degradation, leading to cell malfunctioning and death [57]. HSP90 clients include protein kinases, transcription factors, and receptors critical for maintaining protein homeostasis and regulating vital cellular processes but also proteins linked to various diseases, such as cancer and neurodegenerative disorders [75,76,81,82,83,84]. The pivotal role of HSP90 in sustaining cell signaling and proliferation is thus evident, making it a target to treat proliferative and degenerative diseases, the etiology of which depends on the correct folding of their causative agents. Although no HSP90 inhibitors have yet been approved for clinical use, a wide variety of molecules is currently being developed and investigated [85,86,87,88,89]. The crucial role of HSP90 in chaperoning several important cellular functions required for survival and proliferation of protozoan parasites in the mammalian host makes it a promising drug target for the treatment of various NTDs [20,24,25,26,66,90]. Nevertheless, the identification of new molecules targeting protozoan parasite HSP90 has proven to be a daunting task. The main limitations in drug development are the lack of structural information, especially on full-length proteins, and the conservation of the ATP binding site, determining selectivity issues [91].

### 5.1. Natural Macrocyclic Compounds and Their Derivatives

The most widely studied HSP90 inhibitors are the two macrocyclic natural compounds geldanamycin and radicicol (Table 1), isolated from *Streptomyces hygroscopicus* and *Monosporium bonorden*, respectively [68]. Both compounds are active towards parasite HSP90s, targeting the ATP binding pocket in the NTD (Table 1). Their effects were also investigated in vitro against *Toxoplasma*, *Plasmodium*, and *Trypanosoma* parasites, proving their ability to induce client protein degradation and thus impairing cell viability (Table 1) [21,23,24,25,26,55,67,92,93,94,95,96,97,98,99].

The main limitation to their potential application as anti-parasite drugs is their lack of selectivity, since both macrocycles are able to effectively bind human HSP90 [68,98]. Further problems arise from their poor solubility and significant hepato- and nephrotoxicity [100,101,102]. Despite these adverse properties, geldanamycin was used as a scaffold for the development of derivatives having improved selectivity profiles and reduced side effects [23,96,98,103,104,105]. Few geldanamycin-based compounds have been reported so far, and among those the most active are 17-AAG-GA, 17-AEP-GA, and 17-DMAG-GA (Table 1) [96,103,104].

**Figure 7:**
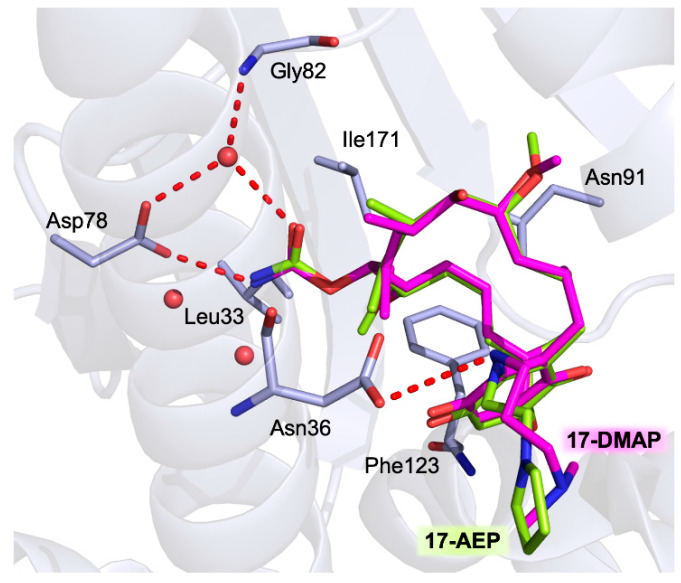
Active-site view of the superimposition between the 17-DMAP-geldanamycin (in sticks, magenta carbons) and 17-AEP-geldanamycin (in sticks, cyan carbons) in complex with *Lm*–NTD (yellow carbons) (PDB ids 3Q5J and 3Q5L, respectively). H-bonds are shown as red dashed lines, and water molecules as red spheres.

The binding modes of 17-AEP-GA and 17-DMAG-GA were unveiled in the complexes with *Lm*-NTD (PDB ids 3Q5J and 3Q5L, respectively) [34]. Both GA-derivatives show the same conformation of the macrocyclic molecular core inside the nucleotide pocket, forming a conserved network of interactions with the surrounding residues (Figure 7). Key H-bonds are entailed with Asp39, Asp78, Gly82, and Phe123 residues playing an important role in substrate binding. The macrocyclic is also stabilized inside the pocket by several van der Walls interactions (Leu33, Leu92, Ile171, Phe123, and Val135). The variable molecular portions of 17-AEP-GA and 17-DMAG-GA (R1, Table 1) point toward the solvent-exposed protein surface. In both structures, the L2 loop of *Lm*-NTD adopts the open state formerly observed in the complex with ADP (Section 3.1).

### 5.2. Other Natural Products and Their Derivatives Acting as Parasite HSP90 Inhibitors

#### 5.2.1. Harmine and Its Derivatives

**Figure 8:**
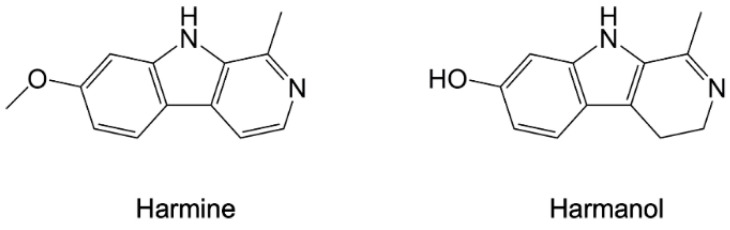
Chemical structures of harmine and harmanol [81,106].

The natural alkaloids harmine and harmanol (Figure 8) were identified as inhibitors of *Pf*-HSP90, showing their ability to target the protein NTD [81] with *K_d_* values of 0.04 mM and 7.0 mM, respectively, as determined by surface plasmon resonance (SPR) [106]. Comparative analysis of the human protein showed a selectivity of harmine towards *Pf*-NTD, whereas harmanol had a higher affinity for the h-NTD (*K_d_* = 0.22 mM). In silico studies performed with both compounds on *Pf* and human NTDs predicted a prominent role of Arg98 in *Pf*-HSP90 for harmine selective binding. This hypothesis was proven by SPR analysis on the R98K mutant of *Pf*-NTD, showing a reduced affinity for the ligand upon arginine replacement by lysine, mimicking the human protein. Harmine also displayed antiplasmodial activity in vitro, reporting synergistic effects with known antimalarial drugs such as chloroquine and artemisinin. Furthermore, in vivo combination studies in mouse models of malaria proved the effectiveness of this compound in improving the potency of chloroquine, without significant toxicity [106,107].

**Figure 9:**
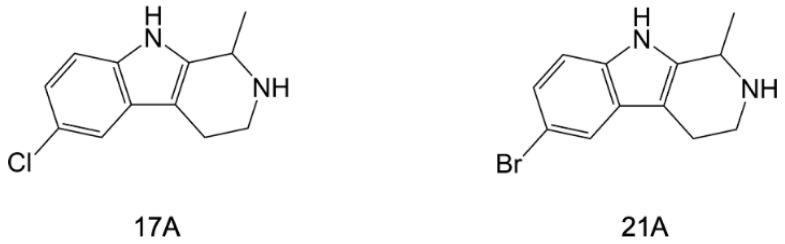
Chemical structures of 17A and 21A [108].

Following these results on harmine, a subsequent study reported the generation of a library of 42 analogues using a microwave-assisted synthetic approach [108]. Among these compounds, 17A and 21A (Figure 9) were the most effective in *Pf*-NTD binding assays with *IC_50_* values of 12.2 (±2.3) µM and 23.1 (±8.8) µM, respectively. The antimalarial activity of 17A and 21A was tested in vitro on *P. falciparum* W2 reporting *IC_50_* values of 4.2 (±1.3) µM and 5.7 (±1.7) µM, respectively, with the latter compound also being effective on artemisinin-resistant strains (*IC_50_* of 9.2 (±0.4) µM and 9.6 (±2.0) µM, on MRA-1236 and MRA-1240 strains, respectively). Further in vitro assays on HepG2 and HeLa cell lines proved the safety profiles of both compounds in the human host. These two harmine analogues were also tested in vivo, showing meaningful antimalarial activity and increased survival in infected BALB/c mice. Addictive effects in combination with dihydro-arthemisinin were reported for 21A, indicating its potential application as a partner-drug candidate.

**Figure 10:**
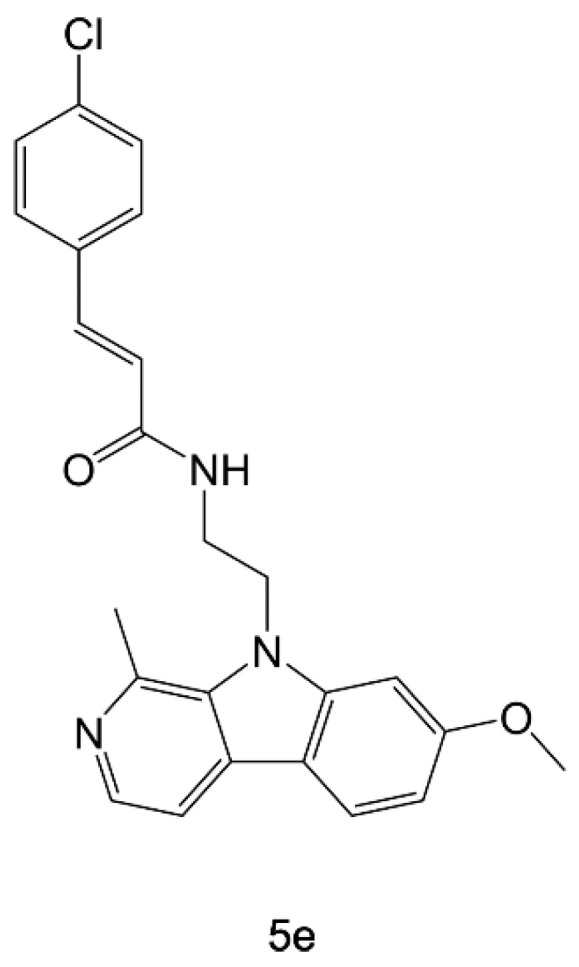
Chemical structure of 5e [109].

In a recent work, harmine analogous have been exploited in combination with cinnamic acid derivatives to develop hybrid compounds, named amide-type harmicines [109]. A library of 16 hybrid molecules was synthesized and tested in vitro against *P. falciparum* chloroquine-sensitive (*Pf*3D7) and chloroquine-resistant (*Pf*Dd2) strains. The most active compound was 5e (Figure 10), having *IC_50_* values of 0.04 µM and 0.17 µM, respectively. Furthermore, cytotoxicity assays of the most active harmicines on human HepG2 cells revealed favorable selectivity indices. The targeting of *Pf*HSP90 was investigated in silico, showing that harmicines bind to the protein NTD.

#### 5.2.2. Antiparasite Phytochemicals and Their Synthetic Derivatives

**Figure 11:**
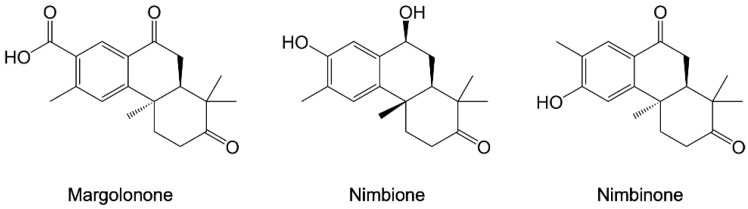
Chemical structures of margolonone, nimbinone, and nimbione [112].

*Azadirachta indica* is a common African plant whose extracts were shown to possess antiplasmodial activity [110,111]. In a recent study, 236 *A. indica* phytochemicals were screened in silico to identify compounds able to target *Pf*-HSP90 with a safe profile, which is essential for their further development as drug candidates [112]. The combination of molecular docking and consensus scoring analysis identified margolonone, nimbinone, and nimbione (Figure 11) as the most effective compounds targeting the nucleotide binding pocket of *Pf*-HSP90.

**Figure 12:**
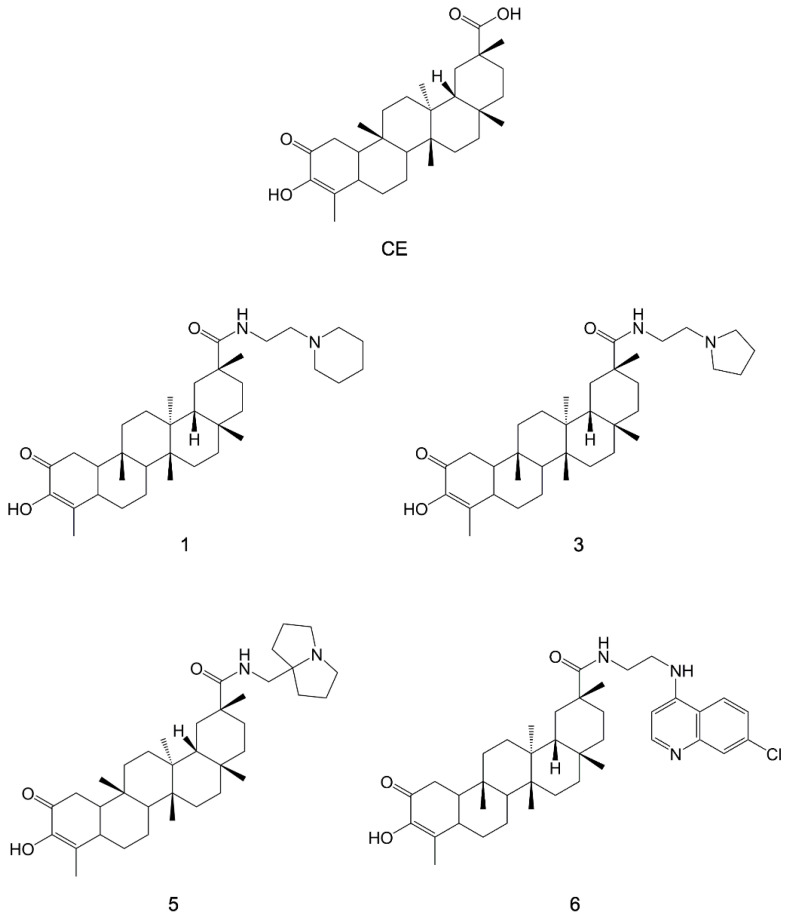
Chemical structures of CE and its carboxamides 1, 3, 5, and 6 [113].

In another study, the natural triterpene celastrol (CE, Figure 12) was used as a lead compound for the design and synthesis of eleven carboxamide derivates tested in vitro for their anti-leishmanial activity [113]. Four CE carboxamides (compounds **1**, **3**, **5**, and **6**; Figure 12) had high cytotoxicity against *L. infantum* and *L. tropica*, having *IC_50_* values in the sub-micromolar range. These compounds also showed a safe profile toward both human (HMEC-1 and THP-1) and murine (BMDM) cells. Compound **3**, the most selective CE analogue of this series, was shown to effectively inhibit *Leishmania* HSP90 ATPase activity in vitro.

### 5.3. Synthetic Parasite HSP90 Inhibitors

**Figure 13:**
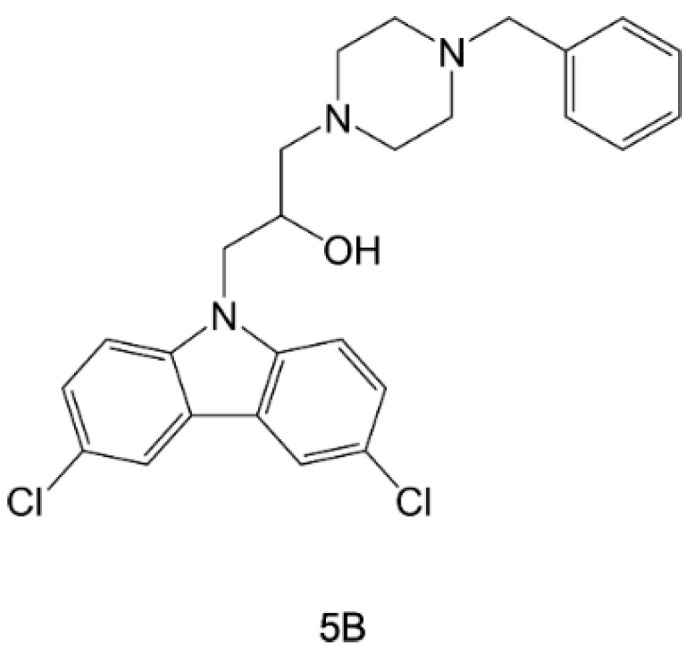
Chemical structure of 5B [114,115].

Virtual docking was applied to a wide set of antimalarial compounds, selected through phenotypic screening, identifying the aminoalcohol carbazole 5B (Figure 13) as a selective *Pf*-HSP90 inhibitor [114,115]. This compound displayed a high binding affinity toward *Pf*-HSP90 (*K_d_* = 28.1 µM, in thermophoresis assays) and cytotoxicity towards *P. falciparum* (*IC_50_* = 82 nM), also having a safe profile (determined on primary rat myoblast L6 cells). In silico studies on 5B predicted its binding to *Pf*-HSP90 in a parasite-specific pocket, accounting for its selective profile.

**Figure 14:**
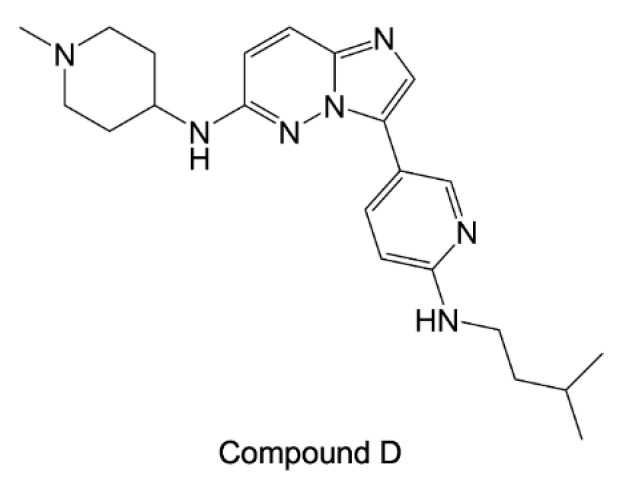
Chemical structure of compound D [116].

A further family of HSP90 inhibitors are imidazopyridazines [116]. A specific subset of these compounds, indicated as class 2 imidazopyridazine, was proposed to target the ATP site of both *P. falciparum* HSP90 and calcium-dependent protein kinase 1 (CDPK1). Among these derivatives, compound D (Figure 14) was shown to effectively bind HSP90 by affinity purification.

**Figure 15:**
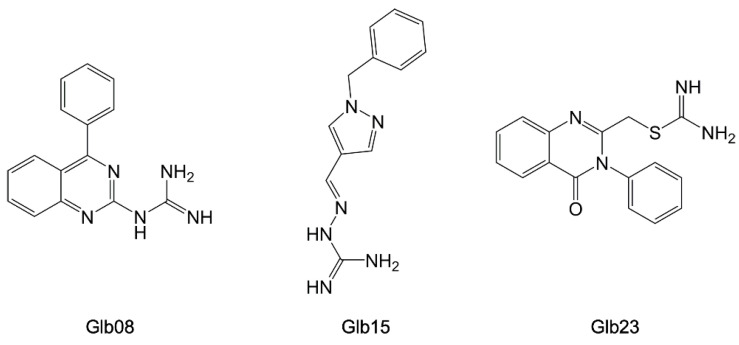
Chemical structures of Gl08, Glb15, and Glb23 [97].

In a following study, the structural information available on *Leishmania* NTD was used for a virtual screening campaign on commercial molecules to discover novel antiparasitic compounds targeting HSP90 [97]; 28 chemically diverse compounds were selected by molecular docking simulations and tested in vitro on both the recombinant *L. braziliensis* HSP90 and *Lb* parasite cells. The three most active compounds of this library resulted in Glb08, Glb15, and Glb23 (Figure 15), inhibiting the ATPase activity of *Lb*-HSP90 at low micromolar concentrations (*IC_50_* = 30 (±1) µM, 36 (±1) µM, and 35 (±1) µM, respectively), and being effective in blocking *L. braziliensis* cell growth.

**Figure 16:**
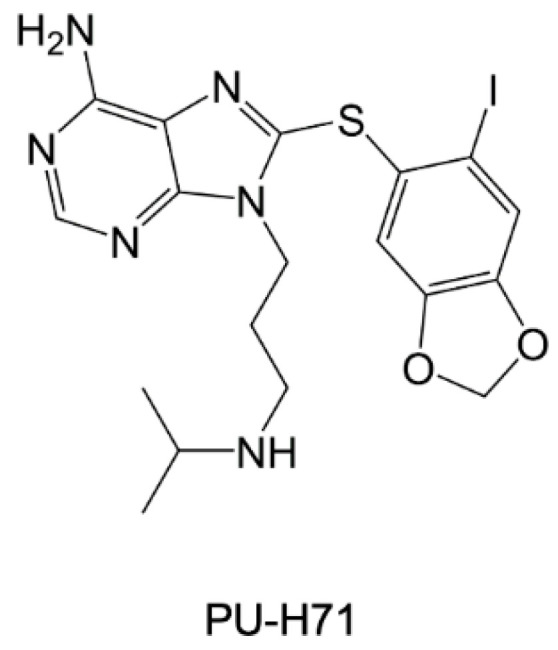
Chemical structure of PU-H71 [117].

The ability of HSP90 to bind nucleotide purine-based compounds has been proposed and investigated for the development of effective inhibitors [32]. The first molecule belonging to this class is PU-H71 (Figure 16), a potent inhibitor *Pf*-HSP90 (*IC_50_* = 511 nM) that showed strong antimalarial properties in vitro [117]. Indeed, PU-H71 exhibited cytotoxic effects on *P. falciparum* (*IC_50_* = 111 nM), displaying also synergistic activity with chloroquine. In vivo studies proved the antiparasitic efficacy of PU-H71 on the BALB/c mouse model of malaria [117].

**Figure 17:**
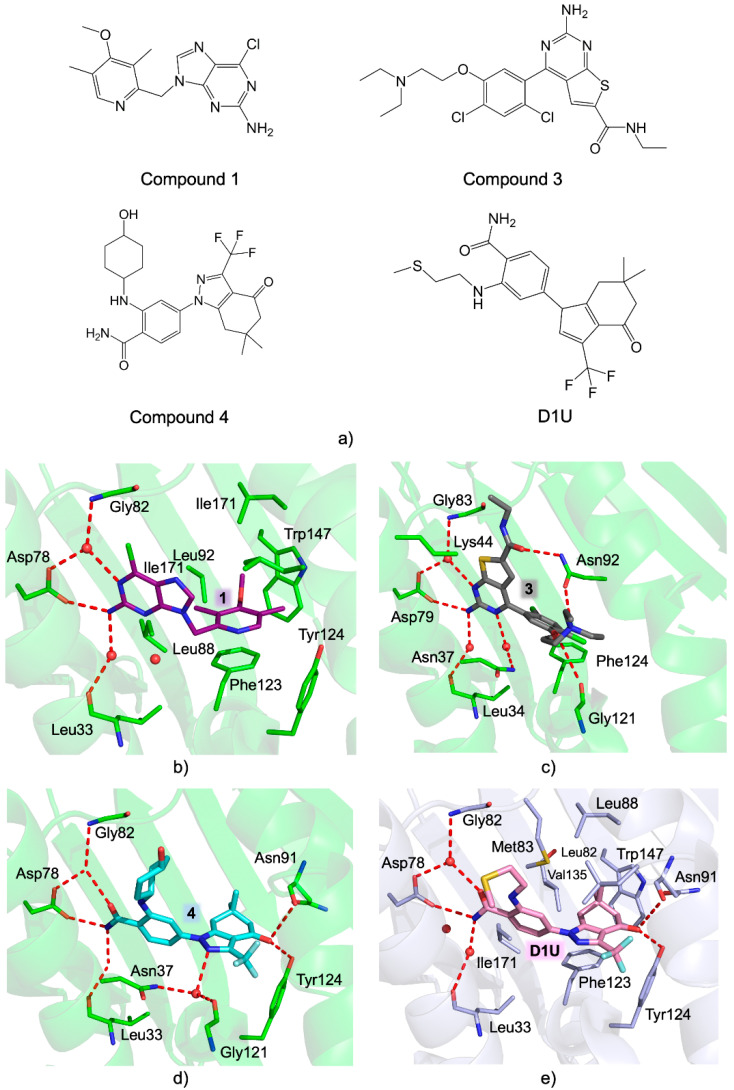
(**a**) Chemical structures of compounds **1**, **3**, **4** [67], and D1U. Active-site view of (**b**–**d**) *Tb*-NTD (green cartoon and carbons) in complex with (**b**) compound **1** (in sticks, dark purple carbons; PDB id 3O6O [67]), (**c**) compound **3** (in sticks, grey carbons; PDB id 3OMU [67]), and (**d**) compound **4** (in sticks, aquamarine carbons; PDB id 3OPD [67]); (**e**) *Lm*-NTD (light blue cartoon and carbons) in complex with D1U (in sticks, pink carbons; PDB id 3Q5K, unpublished results). Sulfur atoms are colored yellow, chlorine light green, and fluorine light cyan.

Two further purine derivatives were reported as *Tb*HSP90 inhibitors, with compound **1** (Figure 17) being the most potent and effective in targeting the NTD of the protein (*K_d_ =* 9 nM, in DSF and ITC assays) [67]. The binding mode of this compound was unveiled in the complex with *Tb*-NTD (PDB code 3O6O [67]). The purine moiety of compound **1** entails the same H-bonds with Asp78 and the three conserved water molecules peculiar also of the complex with ADP (Figure 17). The pyridyl group occupies the hydrophobic pocket stabilized by van der Waals interactions, whereas its aromatic moiety forms a π-stacking interaction with Phe123. Compound **1** resulted in cytotoxic against *T. brucei,* reporting an *EC_50_* = 0.31 µM. In the same study, an analogous compound was reported, namely, the thienopyrimidine derivative compound **3** (Figure 17), displaying a similar affinity for the NTD of *Tb*-HSP90 (*K_d_* = 26 nM) and trypanocidal properties (*EC_50_* = 0.21 µM) [67]. The structure of the complex with *Tb*-NTD (PDB code 3OMU [67]) shows the thienopyrimidine moiety of 3 sharing the same interactions as the purine of 1 (Figure 17). Furthermore, the carboxamide and diethylamino-ethanol moieties of 3 are stabilized within the cavity through a tight network of either direct or water-mediated interactions, with key residues involved also in nucleotide binding.

A further class of parasite HSP90 inhibitors is represented by benzamide-based molecules [32]. Three compounds based on this scaffold were reported as *Tb*-HSP90 inhibitors, showing their ability to target the protein NTD [60]. The most active of this series was compound **4** (*K_d_* = 3 nM), being effective in inhibiting *T. brucei* cell growth (*EC_50_* = 0.10 µM) [60]. The binding mode of 4 in the *Tb*-NTD (PDB code 3OPD [60]) shows its benzamide moiety forming an H-bond with Asp78, a peculiar, conserved nucleotide interaction. The dihydroindazolone and pyrazole groups of 4 entail direct and water-mediated H-bonds inside the HSP90 nucleotide binding cavity.

Benzamide derivatives have been investigated also as inhibitors of *L. major* HSP90. Despite the lack of binding/inhibition data, structural information is available on the complex of the benzamide derivate D1U (Figure 17) with *Lm*-NTD (PBD code 3Q5K, unpublished results). The benzamide moiety of D1U occupies the nucleotide binding pocket, being H-bonded with Asp78 and the three conserved water molecules (Figure 17e). The tetrahydroindazole moiety is accommodated inside the hydrophobic pocket stabilized by van der Waals interactions, with its hydroxyl group establishing an H-bond with Tyr124.

## 6. Conclusions

Protozoan parasites are harmful pathogens responsible for a wide variety of debilitating diseases affecting millions of people worldwide. HSP90 plays a crucial role in the parasite life cycle, since it is essential to overcome the multiple environmental changes occurring in the mammalian host. The NTD of HSP90, performing ATP binding and hydrolysis, has mainly been investigated as a drug target to abolish its chaperone activity. To date, selectivity represents one of the main restrictions for the development of effective antiparasitic drugs targeting HSP90. Notably, structural information on parasite proteins is remarkably limited. To date, only a few structures have been reported on the isolated domains, and no structural information is available on full-length parasite proteins. Thus, more efforts are needed to provide such pivotal information and to deeply investigate the specific functional mechanisms of parasite HSP90s. The current understanding of HSP90 chaperone activity has been recently revised; nonetheless, various transition stages in the mechanism of action are still matters of debate. Although the development of molecules targeting the parasite HSP90-NTD has led to the identification of potent inhibitors, more efforts are required to obtain drug candidates able to selectively target the parasite proteins. The relevant steps forward made in this field and summarized in this review could open new perspectives for the development of innovative, effective, anti-protozoan drugs.

## Figures and Tables

**Table 1:** HSP90 binding and/or inhibition data, and parasite cytotoxicity by natural macrocyclic compounds and their derivatives.

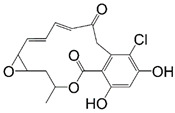 Radicicol		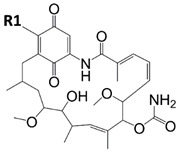 Ansamycin Scaffold	
Compound	R1	HSP Binding and/or Inhibition Data on Parasite HSP90	Parasite Cytotoxicity
Radicicol	-	*Pf*-HSP90 *IC_50_* = 0.01 ug/mL [93] *Lb*-HSP90 *IC_50_* = 80 nM [94]	*T. gondii IC_50_* = 4.9 ug/mL [95] *T. brucei EC_50_* = 70 nM [103]
Geldanamycin	OCH_3_	*Pf*-HSP90 *K_i_* = 2.3 nM [96] *K_d_* = 1.0 µM [23] *Tb*-HSP90 *K_d_* = 0.001 nM [67]	*P. falciparum IC_50_* = 300.0 nM [96], 214.8 nM [104], and 25 nM [23] *P. berghei IC_50_* = 170.0 nM [96] *T. brucei EC_50_* = 13 nM [103] *L. braziliensis IC_50_* = 190 nM [97] *L. donovani IC_50_* = 32 ng/mL [99]
		*Lb*-HSP90 *K_Dapp_* = 7.6 µM [97] *Lb*-HSP90 *IC_50_* = 530 nM [94] *La*-HSP90 *IC_50_* = 190 nM [98]	
17-AEP-GA	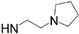	*Tb*-HSP90 *K_d_* = 714 nM [67]	
17-AAG-GA	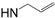	*La*-HSP90 *IC_50_* = 65 nM [105] and 110 nM [98] *Lb*-HSP90 *IC_50_* = 65 nM [105] *Lm*-HSP90 *IC_50_* = 80 nM [105] *Pf*-HSP90 *IC_50_* = 387.5 nM [104]	*P. falciparum IC_50_* = 160 nM [23] *T. brucei EC_50_* = 38 nM [103]
17-DMAG-GA	* 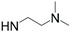 *	*Pf*-HSP90 *K_i_* = 5.7 nM [96] *La*-HSP90 *IC_50_* = 86 nM [98]	*P. falciparum IC_50_* = 170.0 nM [96] and 118.1 nM [104] *P. berghei IC_50_* = 580.0 nM [96] *T. brucei EC_50_* = 3.0 nM [103]

## Data Availability

Data sharing not applicable.

## Supplementary Materials

The following supporting information can be downloaded at: https://www.mdpi.com/article/10.3390/ph15111341/s1, Figure S1: Electron density map detail from Pf-NTD structure reprocessed with PDB-redo; Table S1: HSP90 binding and/or inhibition data and cytotoxicity on parasite and human cells by other natural products and synthetic compounds.

## Abbreviation

NTDs: neglected tropical diseases; HAT: human African trypanosomiasis; T. brucei or Tb: *Trypanosoma brucei*; *T. cruzi* or Tc: *Trypanosoma cruzi*; *L. donovani* or Ld: *Leishmania donovani*; *L. mexicana*: *Leishamania mexicana*; *L. major* or Lm: *Leishmania major*; *P. falciparum* or Pf: *Plasmodium falciparum*; *T. gondii* or Tg: *Toxoplasma gondii*; *A. indica*: *Azadirachta indica*; *L. infantum*: *Leishmania infantum*; WHO: World Health Organization; HSPs: heat shock proteins; sHSP: small-molecular weight heat shock proteins; NTD: N-terminal domain; MD: middle domain; CTD: C-terminal domain; AMP-PNP: adenosine-5-(β,γ-imido)triphosphate; ANP: phosphoaminophosphonic acid-adenylate ester; HepG2: human liver hepatocellular carcinoma cell line; DSF: differential scanning fluorimetry; ITC: isothermal titration calorimetry.
