# Supplementary material for: Structural Basis of Parasitic HSP90 ATPase Inhibition by Small Molecules
Source: Pharmaceuticals (Basel). 2022 Oct 29;15(11):1341. doi: 10.3390/ph15111341 (PMC9692773; doi:10.3390/ph15111341)
Supplement: Supplementary file 1 [file pharmaceuticals-15-01341-s001.zip › pharmaceuticals-1960191-supplementary.pdf]

Structural basis of parasitic HSP90 ATP-ase inhibition by small molecules

Giusy Tassone[1], Marco Mazzorana[2,*] and Cecilia Pozzi[1,*]

[1] Department of Biotechnology, Chemistry and Pharmacy, Department of Excellence 2018–2022, University of Siena, Via Aldo Moro 2, 53100 Siena, Italy
[2] Diamond Light Source Ltd., Diamond House, Harwell Science & Innovation Campus, Didcot, Oxfordshire, OX11 0DE, UK.

* Correspondence: M.M. marco.mazzorana@diamond.ac.uk Tel.: +44 01235 778643; C.P. cecilia.pozzi@unisi.it; Tel.: +39 0577 232132

Table of contents

Figure S1. S2
Table S1. S3
References S9

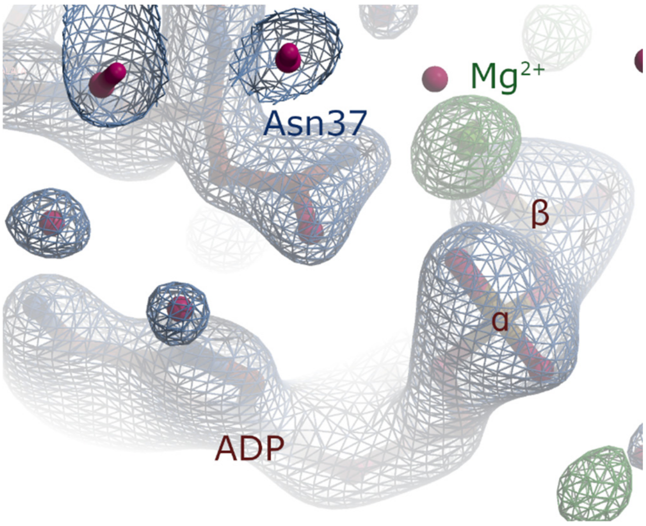

Figure S1. Electron density map detail from *Pf*-NTD structure (PDB id 3K60 [1]) reprocessed with PDB-redo (https://pdb-redo.eu/db/3k60) [2]. The f_0-f_c map (3 σ level) shows a clear positive density not accounted for between the side chain of Asn37 and the α and β phosphates of ADP. The green blob, unaccounted for in the original structure, is compatible with the location of Mg(II) ions in other HSP90 structures.

Table S1. HSP90 binding and/or inhibition data and cytotoxicity on parasite and human cells by other natural products and synthetic compounds.

	Compound	Structure	Parasite HSP90 binding and/or inhibition	Parasite cytotoxicity	Cytotoxicity on human cells
NATURAL COMPOUNDS	Harmine	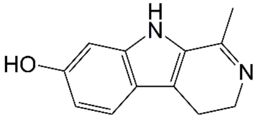	*Pf*-NTD K_d = 0.04 mM [3]	N.A. [3]	N.A. [3]
	Harmanol	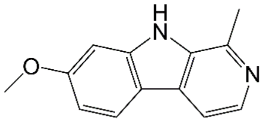	*Pf*-NTD K_d = 7.0 mM [3]	N.A. [3]	N.A. [3]
	17A	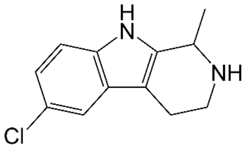	*Pf* IC_{50} = 12.2 ± 2.3 µM [4]	*Pf* W2 IC_{50} = 4.2 ± 1.3 µM [4]	HeLa IC_{50} = 1.1 ± 0.3 mM [4]<br>HepG2 IC_{50} = 0.16 ± 0.01 mM [4]
	21A	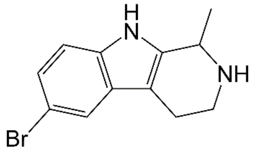	*Pf* IC_{50} = 23.1 ± 8.8 µM [4]	*Pf* W2 IC_{50} = 5.7 ± 1.7 µM [4]<br>*Pf* MRA-1236 = 9.2 ± 0.4 µM [4]<br>*Pf* MRA-1240 = 9.6 ± 2.0 µM [4]	HeLa IC_{50} = 0.48 ± 0.14 mM [4]<br>HepG2 IC_{50} = 0.140 ± 0.001 mM [4]

5e	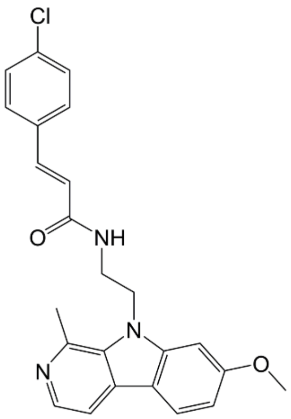	N.A. [5]	*Pf*3D7 IC_{50} = 0.04 ± 0.02 μM [5] *Pf* Dd2 IC_{50} = 0.17 ± 0.01 μM [5]	HepG2 IC_{50} = 2.91 ± 1.75 μM [5]
Margolonone	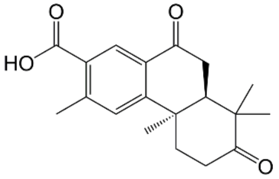	N.A. [6]	N.A. [6]	N.A. [6]
Nimbione	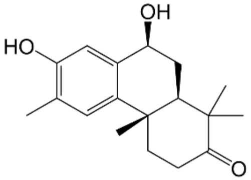	N.A. [6]	N.A. [6]	N.A. [6]
Nimbinone	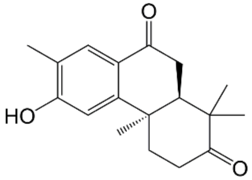	N.A. [6]	N.A. [6]	N.A. [6]

CE	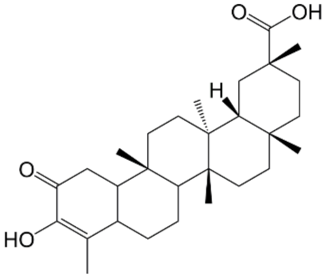	*Lb* IC_{50} = 210 ± 10 μM [7]	*Li* Promatigotes IC_{50} = 0.21 ± 0.07 μM [7]<br>*Lt* Promatigotes IC_{50} = 0.49 ± 0.17 μM [7]	HMEC-1 IC_{50} = 0.38 ± 0.08 μM [7]<br>THP-1 IC_{50} = 0.90 ± 0.30 μM [7]
1	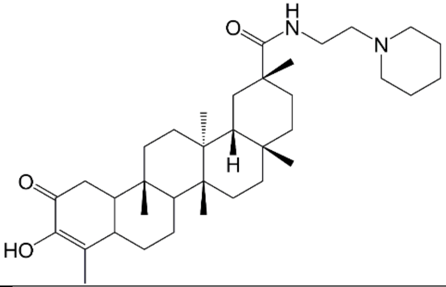	*Lb* IC_{50} = 83 ± 2 μM [7]	*Li* Promatigotes IC_{50} = 0.06 ± 0.02 μM [7]<br>*Lt* Promatigotes IC_{50} = 0.09 ± 0.03 μM [7]<br>*Lt* Amastogotes IC_{50} = 0.19 ± 0.11 μM [7]	HMEC-1 IC_{50} = 0.51 ± 0.08 μM [7]<br>THP-1 IC_{50} = 2.08 ± 0.24 μM [7]
3	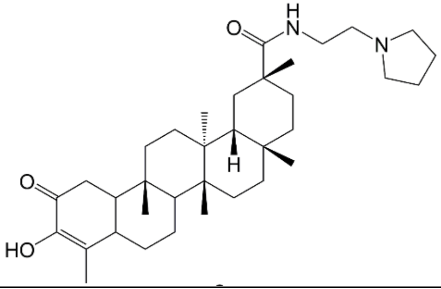	*Lb* IC_{50} = 81 ± 1 μM [7]	*Li* Promatigotes IC_{50} = 0.06 ± 0.01 μM [7]<br>*Lt* Promatigotes IC_{50} = 0.08 ± 0.04 μM [7]<br>*Lt* Amastogotes IC_{50} = 0.13 ± 0.09 μM [7]	HMEC-1 IC_{50} = 0.53 ± 0.17 μM [7]<br>THP-1 IC_{50} = 2.79 ± 0.44 μM [7]
5	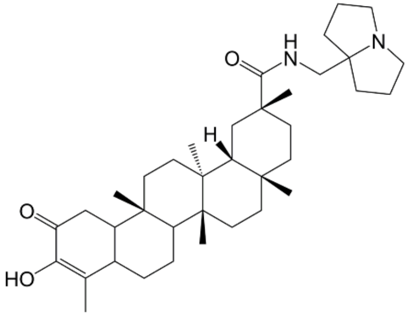	*Lb* IC_{50} = 20 ± 2 μM [7]	*Li* Promatigotes IC_{50} = 0.12 ± 0.02 μM [7]<br>*Lt* Promatigotes IC_{50} = 0.12 ± 0.05 μM [7]<br>*Lt* Amastogotes IC_{50} = 0.52 ± 0.28 μM [7]	HMEC-1 IC_{50} = 0.12 ± 0.03 μM [7]<br>THP-1 IC_{50} = 1.88 ± 0.34 μM [7]

	6	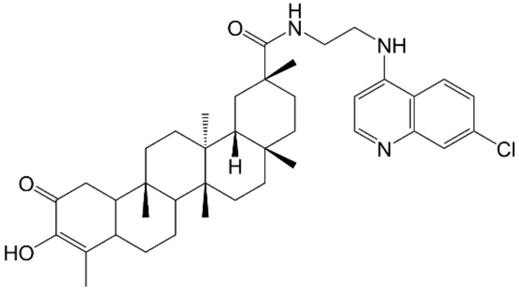	*Lb* IC_{50} = 65 ± 4 μM [7]	*Li* Promatigotes IC_{50} = 0.09 ± 0.03 μM [7]<br>*Lt* Promatigotes IC_{50} = 0.14 ± 0.03 μM [7]<br>*Lt* Amastogotes IC_{50} = 0.66 ± 0.06 μM [7]	HMEC-1 IC_{50} = 0.40 ± 0.03 μM [7]<br>THP-1 IC_{50} = 0.85 ± 0.24 μM [7]
**SYNTHETIC COMPOUNDS**	5B	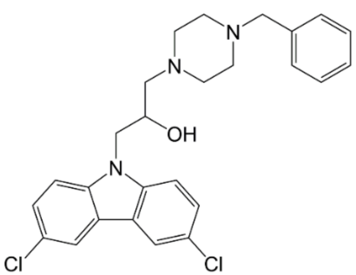	*Pf*-HSP90 K_d = 28.1 μM [8]	*Pf* IC_{50} = 82 nM [8]	N.A. [8]
	Compound D	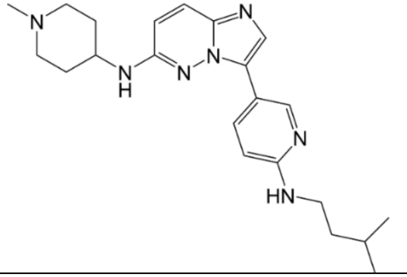	*Pf*-HSP90 K_d = 6.17 μM [9]<br>*Pf*-HSP90-NTD K_d = 10.73 μM [9]	N.A. [9]	N.A. [9]
	Glb08	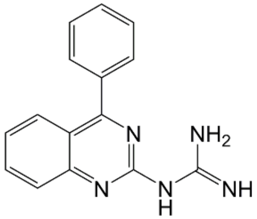	*Lb* IC_{50} = 30 ± 1 μM [10]<br>*Lb*-HSP90 K_{Dapp} = 13 ± 4 μM [10]<br>*Lb*-NTD K_{Dapp} = 17 ± 6 μM [10]	*Lb* IC_{50} = 43 ± 2 μM [10]	N.A. [10]

Glb15	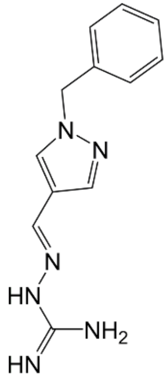	*Lb* IC_{50} = 36 ± 1 μM [10]<br>*Lb*-HSP90 K_{Dapp} = 14 ± 4 μM [10]<br>*Lb*-NTDK_{Dapp} = 13 ± 3 μM [10]	*Lb* IC_{50} = 71 ± 2 μM [10]	N.A. [10]
Glb23	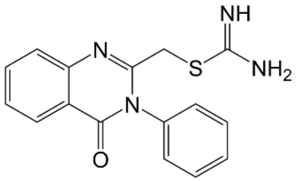	*Lb* IC_{50} = 35 ± 1 μM [10]<br>*Lb*-HSP90 K_{Dapp} = 26 ± 7 μM [10]<br>*Lb*-NTDK_{Dapp} = 20 ± 10 μM [10]	*Lb* IC_{50} = > 100 μM [10]	N.A. [10]
PU-H71	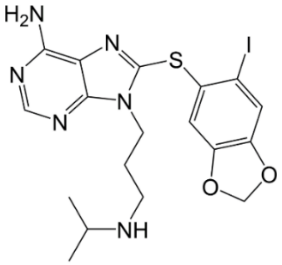	*Pf*-NTD K_d = 70.8 ± 0.006 μM [11]	*Pf* IC_{50} = 111 nM [11]	N.A. [11]
Compound 1	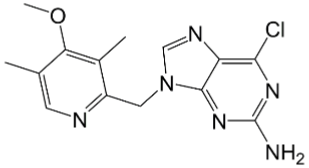	*Tc*-NTD K_d = 9 ± 2 nM [12]	*Tc* IC_{50} = 0.31 μM [12]	N.A. [12]
Compound 3	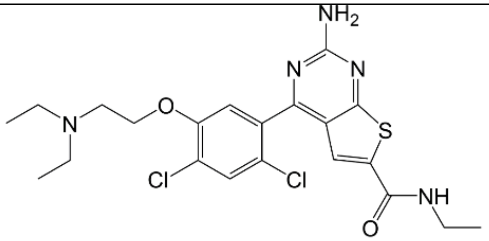	*Tc*-NTD K_d = 26 ± 9 nM [12]	*Tc* IC_{50} = 0.21 μM [12]	N.A. [12]

Compound 4	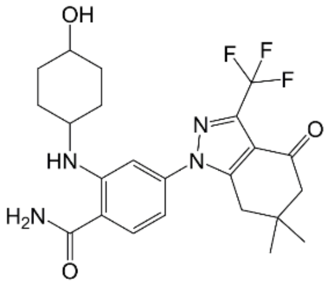	*Tc*-NTD K_d = 3 ± 1 nM [12]	*Tc* IC_{50} = 0.10 μM [12]	N.A. [12]
D1U	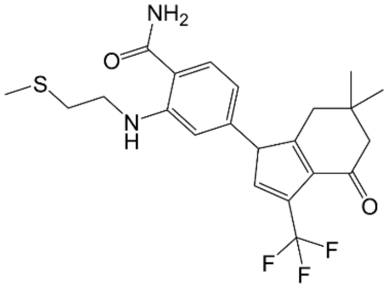	N.A.	N.A.	N.A.

N.A = Not Available

References

1. Corbett, K.D.; Berger, J.M. Structure of the ATP-Binding Domain of Plasmodium Falciparum Hsp90. *Proteins* **2010**, *78*, 2738–2744, doi:10.1002/prot.22799.

2. Joosten, R.P.; Long, F.; Murshudov, G.N.; Perrakis, A. The PDB_REDO Server for Macromolecular Structure Model Optimization. *IUCrJ* **2014**, *1*, 213–220, doi:10.1107/S2052252514009324.

3. Shahinas, D.; Macmullin, G.; Benedict, C.; Crandall, I.; Pillai, D.R. Harmine Is a Potent Antimalarial Targeting Hsp90 and Synergizes with Chloroquine and Artemisinin. *Antimicrob Agents Chemother* **2012**, *56*, 4207–4213, doi:10.1128/AAC.00328-12.

4. Bayih, A.G.; Folefoc, A.; Mohon, A.N.; Eagon, S.; Anderson, M.; Pillai, D.R. In Vitro and in Vivo Anti-Malarial Activity of Novel Harmine-Analog Heat Shock Protein 90 Inhibitors: A Possible Partner for Artemisinin. *Malar J* **2016**, *15*, 579, doi:10.1186/s12936-016-1625-7.

5. Marinović, M.; Perković, I.; Fontinha, D.; Prudêncio, M.; Held, J.; Pessanha de Carvalho, L.; Tandarić, T.; Vianello, R.; Zorc, B.; Rajić, Z. Novel Harmicines with Improved Potency against Plasmodium. *Molecules* **2020**, *25*, doi:10.3390/molecules25194376.

6. Daniyan, M.O.; Ojo, O.T. In Silico Identification and Evaluation of Potential Interaction of Azadirachta Indica Phytochemicals with Plasmodium Falciparum Heat Shock Protein 90. *Journal of Molecular Graphics and Modelling* **2019**, *87*, 144–164, doi:10.1016/j.jmgm.2018.11.017.

7. Bassanini, I.; Parapini, S.; Ferrandi, E.E.; Gabriele, E.; Basilico, N.; Taramelli, D.; Sparatore, A. Design, Synthesis and In Vitro Investigation of Novel Basic Celastrol Carboxamides as Bio-Inspired Leishmanicidal Agents Endowed with Inhibitory Activity against Leishmania Hsp90. *Biomolecules* **2021**, *11*, doi:10.3390/biom11010056.

8. Wang, T.; Mäser, P.; Picard, D. Inhibition of Plasmodium Falciparum Hsp90 Contributes to the Antimalarial Activities of Aminoalcohol-Carbazoles. *J Med Chem* **2016**, *59*, 6344–6352, doi:10.1021/acs.jmedchem.6b00591.

9. Green, J.L.; Moon, R.W.; Whalley, D.; Bowyer, P.W.; Wallace, C.; Rochani, A.; Nageshan, R.K.; Howell, S.A.; Grainger, M.; Jones, H.M.; et al. Imidazopyridazine Inhibitors of Plasmodium Falciparum Calcium-Dependent Protein Kinase 1 Also Target Cyclic GMP-Dependent Protein Kinase and Heat Shock Protein 90 To Kill the Parasite at Different Stages of Intracellular Development. *Antimicrob Agents Chemother* **2015**, *60*, 1464–1475, doi:10.1128/AAC.01748-15.

10. Batista, F.A.H.; Ramos, S.L.; Tassone, G.; Leitão, A.; Montanari, C.A.; Botta, M.; Mori, M.; Borges, J.C. Discovery of Small Molecule Inhibitors of Leishmania Braziliensis Hsp90 Chaperone. *J Enzyme Inhib Med Chem* **2020**, *35*, 639–649, doi:10.1080/14756366.2020.1726342.

11. Shahinas, D.; Folefoc, A.; Taldone, T.; Chiosis, G.; Crandall, I.; Pillai, D.R. A Purine Analog Synergizes with Chloroquine (CQ) by Targeting Plasmodium Falciparum Hsp90 (PfHsp90). *PLoS ONE* **2013**, *8*, e75446, doi:10.1371/journal.pone.0075446.

12. Pizarro, J.C.; Hills, T.; Senisterra, G.; Wernimont, A.K.; Mackenzie, C.; Norcross, N.R.; Ferguson, M.A.J.; Wyatt, P.G.; Gilbert, I.H.; Hui, R. Exploring the Trypanosoma Brucei Hsp83 Potential as a Target for Structure Guided Drug Design. *PLOS Neglected Tropical Diseases* **2013**, *7*, e2492, doi:10.1371/journal.pntd.0002492.
